# Relationships between Maternal Folic Acid Supplementation and *GATA4* Gene Polymorphisms in Patients with Non-Chromosomal Congenital Heart Disease: A Hospital-Based Case–Control Study in China

**DOI:** 10.3390/nu15204478

**Published:** 2023-10-23

**Authors:** Letao Chen, Tubao Yang, Tingting Wang, Mengting Sun, Jiabi Qin

**Affiliations:** 1 Infection Control Center, Xiangya Hospital, Central South University, Changsha 410017, China; chenletao93@csu.edu.cn; 2 National Clinical Research Center for Geriatric Disorders, Xiangya Hospital, Central South University, Changsha 410017, China; 3 Department of Epidemiology and Health Statistics, Xiangya School of Public Health, Central South University, Changsha 410017, China; yangtb@csu.edu.cn (T.Y.); 226901004@csu.edu.cn (M.S.)

**Keywords:** folic acid supplementation, *GATA4*, congenital heart disease, gene–environment interactions

## Abstract

This study aimed to investigate the relationships between maternal FA supplementation and nine single-nucleotide variants of the *GATA4* gene in non-chromosomal CHD and further explore the gene–environment interactions associated with CHD. A total of 585 CHD patients and 600 controls were recruited in the case–control study. Maternal FA (FA-containing multivitamin) supplementation information and nine polymorphisms of the *GATA4* gene were collected in this study. Adjusted ORs (aOR) and their 95% confidence intervals (CIs) were calculated using proper statistical methods to analyze the relationships between the two main exposures of interest with respect to CHD. After adjusting the suspicious confounding factors, a significantly increased risk for CHD in offspring was found with non-FA supplementation before/during the pregnancy to CHD in offspring (aOR = 1.58, 95% CI: 1.01–2.48). We suggested taking FA supplementation before/during the pregnancy to prevent CHD in offspring, especially in the preconception period (aOR = 0.53, 95% CI: 0.32–0.90). The genetic results showed that the polymorphisms of rs4841588, rs12458, and rs904018 under specific genotypes and genetic models were significantly related to CHD. The gene–environment interaction between rs10108052 and FA supplementation before/during pregnancy could increase the risk of CHD (aOR = 5.38, 95% CI: 1.67–17.09, *P_interaction_* = 0.004). Relationships between maternal FA supplementation and specific polymorphisms of the *GATA4* gene, as well as the gene–environment interaction, were significantly associated with CHD in offspring.

## 1. Introduction

Congenital heart disease (CHD) is the most common type of birth defect and is the main cause of morbidity and mortality due to congenital defects. The overall prevalence has been estimated at 9.4 per 1000 live births worldwide [1]. Despite the improvement in surgical and clinical management, millions of newborns are affected by CHD every year [2]. The etiology of non-chromosomal CHD is complicated and multifactorial and mainly caused by a combination of genetic and environmental factors [3].

The transcription factor *GATA4*, as the predominant GATA family member, plays a key role in regulating embryonic cardiac development, is the critical modifier in the early stages of cardiac formation, and contributes to the development of the myocardium, endocardium, and conduction system [4]. Evidence has shown that a dysfunction of the *GATA4* gene could increase the risk of CHD. The genotype and allele distributions of the polymorphisms at the *GATA4* gene (such as rs867858, rs904018, and rs884662) are significantly different between CHD cases and controls and are associated with CHD [5,6]. Although the single-nucleotide variations in the *GATA4* gene are firmly considered risk factors, studies have yielded conflicting results regarding specific mutations [7]. The disagreements across studies might be related to diverse populations, sample sizes, selection bias, and a lack of consideration of gene–environment interactions.

Maternal folic acid (FA) supplementation around the periconceptional period is generally established to prevent neural tube defects. Animal studies have indicated that FA deficiency might negatively affect the migration of the cardiac neural crest in the development of the embryonic heart and the subsequent formation of the cardiovascular system, but the precise role in CHD was still deficient and inconsistent [8]. Studies from Asia and Europe were more likely to support the preventive effect of FA supplementation on CHD [9,10,11]; otherwise, inconclusive results have been found in North America [12,13]. Xu et al. conducted a meta-analysis which included 20 original epidemiological studies to assess the correlation between maternal FA supplementation and CHD, and a significant decrease in risk was found [14]. Geographical heterogeneity was observed across the eligible studies, and it is worth noting that the risk in Chinese and European populations was more likely to be reduced by maternal FA supplementation.

To date, the FA–gene interactions associated with CHD are not fully understood, and folate-metabolizing genes have primarily been taken into account in previous studies. The *MTHFR*, *MTR*, and *MTRR* genes were found to be essential to FA cycle metabolism, and most studies have focused on the association of these folate-metabolizing genes with CHD [12,15]. However, a genome-wide association study (GWAS) showed that several newly identified folate-regulatory genes were related to plasma folate concentration, indicating that the genes associated with folate metabolism should be expanded [16,17]. Few studies have investigated the potential interactions between FA-related environmental factors and *GATA4* polymorphisms, and relevant studies could connect the dots regarding CHD risk factors and reveal the mechanisms of CHD. Therefore, the purpose of this case–control study was to investigate the relationships between maternal FA supplementation and single-nucleotide polymorphisms (SNPs) of the *GATA4* gene in non-chromosomal CHD and further explore the gene–environment interactions associated with CHD.

## 2. Materials and Methods

### 2.1. Study Participants

The participants in this study were recruited in Hunan Children’s Hospital (Changsha, China) between December 2018 and June 2021. A total of 585 unrelated CHD patients and 600 healthy individuals with ages ranging from days to 1 year were included. The CHD cases were all non-chromosomal CHD inpatients diagnosed by professional clinicians according to the ICD–10 (International Classification of Diseases, Tenth Revision), while those with chromosomal aberrations, syndrome CHD, or other congenital malformations were excluded. The controls were randomly selected from the healthy individuals without any congenital defects or cardiac disease in the children’s health care department. To minimize the genetic confounding factors, CHD cases and controls with any familial relationships were excluded. A sample of 3 mL of peripheral venous blood was collected from each individual, and maternal pregnancy information was obtained after permission was granted. Ethical consent was given by the ethics committee at the Xiangya School of Public Health, Central South University, in January 2018 (No. XYGW-2018-36).

### 2.2. Data/Sample Collection and Management

Maternal pregnancy information was collected via face-to-face investigation using structured questionnaires among the CHD and control groups. The standardized questionnaire included the following information: (1) demographic characteristic information (e.g., average pregnancy age and annual household income); (2) history of pregnancy and delivery (e.g., adverse pregnancy outcome); (3) history of disease (e.g., diabetes, hypertension, and influenza); and (4) personal lifestyle (e.g., smoking and drinking) before and during the pregnancy. Blood samples were collected and restored in an ultra-low-temperature refrigerator temporarily and sent to BoMiao Biological Technology (Beijing, China) for DNA extraction and genotyping via the MassArray sequencing technique.

### 2.3. Main Exposure and Covariates

Maternal FA (FA-containing multivitamin) supplementation and polymorphisms of the *GATA4* gene were the two main interested forms of exposure in this study. Exposure information on maternal FA supplementation was evaluated via the initiation of supplement timing before/during the pregnancy for three time windows: preconception period (−12 to −1 weeks), the first trimester of pregnancy (0 to 12 weeks), and the middle/last trimesters of pregnancy (13 weeks to the conception). Single-nucleotide variants of the *GATA4* gene were selected by screening the mutation variants in CHD patients in previous studies and searching the dbSNP database of NCBI (https://www.ncbi.nlm.nih.gov/SNP/), accessed on 15 May 2022. Eventually, nine common variants (rs4841588, rs884662, rs804287, rs3203358, rs867858, rs2645457, rs10108052, rs12458, and rs904018) of the *GATA4* gene with minor allele frequency (MAF) ≥ 0.05 were included in this study.

### 2.4. Statistic Analysis

The distributions of baseline characteristics and genetic frequencies between the CHD and control groups were assessed using chi-squared tests. The Hardy–Weinberg equilibrium (HWE) and FDR adjustment were performed in the control group to test the representativeness of the population. The relationships between single-nucleotide variants of the *GATA4* gene and CHD were calculated using genotype frequency distributions and genetic models (dominant, additive, and recessive). Genetic models were categorized into dominant models (“heterozygous + mutant” vs. wild type), additive models (“heterozygous vs. wild type” and “mutant vs. wild type”), and recessive models (mutant vs. “wild type + heterozygous”). FA supplementation was classified by two binary variables: took FA supplementation before or during pregnancy and took FA supplementation in the preconception period; the genetic variables of target loci defaulted to dominant models in the gene–environment interaction analyses. The associations between maternal FA supplementation and SNPs of target loci at the *GATA4* gene and the gene–environment interactions with CHD were estimated by calculating the adjusted ORs (aORs) and their 95% confidence intervals (CIs) from unconditional logistic regression analyses. All statistical data analyses were performed using SPSS version 26.0 (SPSS Inc., Chicago, IL, USA) and R version 4.2.1. All tests were performed using the two-sided approach and significance was considered at *p* < 0.05.

## 3. Results

### 3.1. Maternal Baseline Characteristics

The distributions of basic characteristics between 585 CHD cases and 600 controls are listed in Table 1. Statistical differences were observed in the distributions of pregnancy age, economic status, history of adverse pregnancy, pre-gestational diabetes, gestational diabetes, gestational hypertension, influenza before/during pregnancy, smoking before/during pregnancy, passive smoking before/during pregnancy, and alcohol intake before/during pregnancy in the CHD and control groups (*p* < 0.05). The mothers in the CHD group were more likely to have a lower household income, to experience adverse pregnancy outcomes, to be affected by pregnancy complications, and to have a bad lifestyle before/during the pregnancy compared with the mothers in the control group.

### 3.2. Associations between Maternal FA Supplementation and CHD

We found that about 11% of the participants had never taken any FA supplementation before or during the pregnancy, and the majority of the mothers were supplemented in the first trimester of the pregnancy period (Table 2). After adjusting the suspicious confounding factors, a significantly increased risk of CHD in offspring was found among non-FA-supplementation mothers (aOR = 1.58, 95% CI: 1.01–2.48). We further classified the FA supplementation into three time periods. The unadjusted associations were statistically significant between FA supplementation in the preconception period (cOR = 0.29, 95% CI: 0.19–0.45) and the first trimester of pregnancy (cOR = 0.50, 95% CI: 0.34–0.74) with CHD. Adjustment for the confounding factors showed that FA supplementation in the preconception period (aOR = 0.53, 95% CI: 0.32–0.90) was significantly and protectively associated with CHD.

### 3.3. Associations between Polymorphisms of Target Loci at the GATA4 Gene and CHD

The HWE test showed that eight SNPs followed the HWE after the FDR adjustment (*Q_FDR_* > 0.05), except for rs867858 (Table 3). The comparisons of genotypes and genetic models of target loci at the *GATA4* gene between the CHD and control group are summarized in Table 4. After adjusting for the confounding factors, the polymorphisms of rs4841588 (GG/TT: aOR = 0.41, 95% CI: 0.24–0.70; additive model: aOR = 0.77, 95% CI: 0.62–0.95; recessive model: aOR = 0.41, 95% CI: 0.25–0.69), rs12458 (AA/GG: aOR = 2.20, 95% CI: 1.50–3.23; AG/GG: aOR = 1.87, 95% CI: 1.34–2.60; dominant model: aOR = 1.98, 95% CI: 1.45–2.69; additive model: aOR = 1.49, 95% CI: 1.23–1.81; recessive model: aOR = 1.48, 95% CI: 1.08–2.04), and rs904018 (additive model: aOR = 0.81, 95% CI: 0.66–0.99) of the *GATA4* gene were significantly related to CHD (*p* < 0.05).

### 3.4. Interactions between the Polymorphisms of the GATA4 Gene and FA Supplementation with CHD

The effects of potential interactions between target loci at the *GATA4* gene and FA supplementation with CHD were analyzed (Table 5). After adjusting for the confounding factors, the interaction between rs10108052 (aOR = 5.38, 95% CI: 1.67–17.09, *P_interaction_* = 0.004), and FA supplementation taken before/during pregnancy was found. The interactions between other comparisons were not associated with CHD.

## 4. Discussion

In this study, we investigated the relationships between maternal FA supplementation and nine polymorphisms of the *GATA4* gene and the gene–environment interactions with CHD in 585 CHD patients and 600 healthy subjects from Chinese population. After adjusting for the confounding factors, a significantly increased risk for CHD in offspring was found with non-FA supplementation before/during pregnancy, which meant the data provided support for the hypothesis that maternal FA supplementation would decrease the risk of CHD in offspring. The initiation of FA supplementation timing was optimum in the preconception period (−12 to −1 weeks). The analyses of polymorphisms at the *GATA4* gene associated with CHD showed that the polymorphisms of rs4841588, rs12458, and rs904018 under specific genotypes and genetic models were significantly related to CHD. In addition, we explored the gene–environmental interactions, which indicated that the synergistic interaction between the rs10108052 polymorphism and FA supplementation might increase the risk of CHD in offspring.

According to our results, it is suggested to supplement FA before pregnancy to prevent CHD in offspring. However, the findings were inconsistent with whether maternal FA supplementation would reduce the risk of CHD in offspring among studies in different geographical areas and with different study designs [14]. Studies from China have been more likely to prove the preventive effect of FA supplementation on CHD, but inconsistent results have been found in other countries. Mao showed that maternal FA supplementation before the pregnancy caused a 58% reduction in CHD based on a birth cohort study in Gansu Province (China) [18], which was consistent with our study. Qu conducted a large-sample case-control study including 8379 CHD cases and 6918 controls in Guangzhou Province (China), showing a significant protective association (aOR = 0.69, 95% CI: 0.62–0.76) between first-trimester maternal FA supplementation and CHD [19]. Similar results were observed in other studies of the Chinese population, as well as in Budapest (Hungary) and Atlanta (America) [20,21], which indicated that maternal FA supplementation before/during pregnancy would reduce the risk of CHD in offspring. However, null significant associations were found in the two unique large prospective birth cohorts from Denmark and Norway [22]. Several case–control studies from North America did not find any preventive effect of maternal FA supplementation before/during pregnancy with CHD after the policy of FA food fortification.

The discrepancy of results among different geographical areas might be mainly caused by the diet habits of different countries. The traditional Chinese diet has a lower intake of folate-rich food than that in the West. Since 1993, the Ministry of Health of China has been recommending that women who plan to be pregnant take 0.4 mg of FA tablets every day in order to prevent neural tube defects [23]. As far as we know, there are still some women who have never taken any FA supplementation before or during their pregnancy. This might be explained by unplanned pregnancies, the weak awareness of antenatal care, or the lack of publicity of health knowledge. In Canada, the United States, and other foreign countries, the FA fortification of staple food (mainly flour, pasta, and cornmeal) was mandatory in 1998, which might have improved circulating folate levels [24,25]. Liu found that the birth prevalence of CHD showed a temporal pattern of declining after the introduction of FA food fortification and confirmed that FA food fortification was related to a decreased risk of non-chromosomal CHD [26]. In the population with high circulating folate levels, the ineffective results between FA supplementation and CHD were more likely to be reported.

The *GATA4* gene, as one of the most important transcription factors, is considered to be crucial for regulating cardiac formation. The irregular mutation of the *GATA4* gene is related to CHD, but the regulatory mechanism of the *GATA4* factor remains unclear. The genetic distributions of rs4841588 were opposite to those in the case–control study by Huang in the Chinese population [27]. The study by Li [6] identified that rs884662, and rs3203358 had a strong correlation with CHD, whereas these two SNPs did not show any positive effect on CHD in our present study. These inconsistencies might be related to the different sample source. To date, few studies have evaluated the associations with single-nucleotide variations in the *GATA4* gene and CHD under specific genetic models. The relationships with rs4841588 under additive and recessive models, rs12458 under additive and recessive models, and rs904018 under additive models are significantly related to CHD and similar to the genotype distributions. Therefore, we suggest that, in China, rs12458 might contribute to an increased risk of CHD, and rs4841588 and rs904018 might have a protective effect on CHD.

The occurrence of CHD might be caused by a combination of genetic variations and maternal lifestyle factors before or during pregnancy. Gene–environment interactions could be deterministic for CHD in individuals who were exposed to environmental risk factors [28]. Recent studies have suggested that the genes related to FA metabolism (i.e., *MTHFR*, *MTRR*, *MTR,* and *CBS*) might have gene–environment interactions that influence individual susceptibility to CHD [29,30]. In addition to the genes that directly regulate folate metabolism, several lines of evidence based on GWAS suggest that other genes (i.e., *SYT9*, *FIGN,* and *NBPF3*) are linked to FA concentration [17,31]. Therefore, the exploration of genetic variations with FA should be expanded. In our study, the interaction between rs10108052 and maternal FA supplementation before or during pregnancy could significantly increase the risk of CHD; thus mothers with rs10108052 of the *GATA4* gene are actively recommended to undergo FA supplementation in the preconception period.

### Study Strengths and Limitations

We recruited a case–control study to investigate the relationships between maternal FA supplementation and single-nucleotide variants of the *GATA4* gene in non-chromosomal CHD and further explored the gene–environment interactions associated with CHD. In this study, environmental factors and genetic factors of 1185 individuals were collected to explain the relationships with CHD. The main exposure of FA supplementation was classified according to the initiation of supplement use before/during the pregnancy to evaluate the effect of FA supplementation timing on CHD. The genetic distributions of nine SNPs of the *GATA4* gene were estimated under specific genotypes and genetic models. As far as we know, this is the first study to explore the interactions between the SNPs of the *GATA4* gene and maternal FA supplementation and CHD.

The present study still has several limitations. First, we only collected information on whether the mothers were undergoing FA supplementation and on the timing of supplementation initiation (before or during the conception period). The information about FA intake on the individual level and dietary FA was deficient. Second, the relationships between the risk factors and specific CHD subtypes were not analyzed due to the sample size. Third, the retrospective design of our study might have led to recall bias, which could have affected the accuracy of the data collection. Therefore, there is still space to investigate the effect of maternal FA supplementation and SNPs of the *GATA4* gene and their interactions with CHD.

## 5. Conclusions

In this study, protective relationships were found between maternal FA supplementation and CHD, and specific polymorphisms of the *GATA4* gene were associated with CHD in different genetic models. Furthermore, we evaluated the effect of interactions between maternal FA supplementation and *GATA4* genetic polymorphisms, which indicated that the exploration of valuable gene–environment interactions between FA supplementation and genes in CHD has to be expanded. Since maternal exposure to related environmental factors may have synergistic or antagonistic interactions with specific gene loci, pregnancy health care—reducing maternal exposure to adverse factors and carrying out perinatal health examinations (including undergoing sufficient FA supplementation and gene sequencing)—is necessary for reducing the risk of congenital disease in offspring.

## Figures and Tables

**Table 1:** Maternal baseline characteristics of CHD cases and controls.

Maternal Baseline Characteristics	CHD Cases (*n* = 585)		Controls (*n* = 600)		*χ* ^2^	*p* Value
	*n*	%	*n*	%		
Pregnancy age (years)					11.014	0.004 *
≤24	164	28.0	119	19.8		
25–29	223	38.1	251	41.8		
≥30	198	33.9	230	38.4		
Average annual household income (RMB)					302.674	<0.001 *
≤50,000	463	79.1	174	29.0		
50,001–100,000	88	15.0	264	44.0		
100,001–150,000	12	2.1	51	8.5		
>150,000	22	3.8	111	18.5		
BMI before pregnancy (kg/m^2^)					2.345	0.506
<18.5	111	19.0	115	19.2		
18.5–23.9	390	66.6	380	63.3		
24.0–27.9	60	10.3	75	12.5		
≥28.0	24	4.1	30	5.0		
History of adverse pregnancy					15.844	<0.001 *
No	258	44.1	334	55.7		
Yes	327	55.9	266	44.3		
Pre-gestational diabetes					20.358	<0.001 *
No	523	89.4	577	96.2		
Yes	62	10.6	23	3.8		
Gestational diabetes					12.785	<0.001 *
No	526	89.9	572	95.3		
Yes	59	10.1	28	4.7		
Pre-gestational hypertension					0.139 ^#^	0.750
No	580	99.1	596	99.3		
Yes	5	0.9	4	0.7		
Gestational hypertension					22.077	<0.001 *
No	532	90.9	584	97.3		
Yes	53	9.1	16	2.7		
Influenza before/during pregnancy					34.304	<0.001 *
No	331	56.6	437	72.8		
Yes	254	43.4	163	27.2		
Smoking before/during pregnancy					19.807	<0.001 *
No	533	91.1	583	97.2		
Yes	52	8.9	17	2.8		
Passive smoking before/during pregnancy					33.243	<0.001 *
No	270	46.2	377	62.8		
Yes	315	53.8	223	37.2		
Alcohol intake before/during pregnancy					35.033	<0.001 *
No	459	78.5	545	90.8		
Yes	126	21.5	55	9.2		

BMI, body mass index. * *p* < 0.05, the difference between the CHD cases and controls is significant. ^#^ Using Fisher’s exact test.

**Table 2:** Association between FA supplementation and CHD.

	CHD Cases (*n* = 585)		Controls (*n* = 600)		cOR (95% CI)	*p*	aOR (95% CI) ^#^	*p*
	*n*	%	*n*	%				
FA supplementation before or during pregnancy								
Yes	499	85.3	556	92.7	1.00	-	1.00	-
No	86	14.7	44	7.3	2.18 (1.49–3.19)	<0.001 *	1.58 (1.01–2.48)	0.044 *
Initiation of FA supplementation								
None	86	14.7	44	7.3	1.00	-	1.00	-
The preconception period	87	14.9	154	25.7	0.29 (0.19–0.45)	<0.001 *	0.53 (0.32–0.90)	0.018 *
The first trimester of pregnancy	387	66.1	397	66.2	0.50 (0.34–0.74)	<0.001 *	0.64 (0.41–1.01)	0.056
The middle/last trimesters of pregnancy	25	4.3	5	0.8	2.56 (0.92–7.14)	0.073	1.66 (0.56–4.97)	0.362

cOR indicates crude odds ratios based on univariate analysis; aOR indicates adjusted odds ratios based on multivariable logistic regression. * *p* < 0.05; the difference between case and control is significant. ^#^ Adjusted for pregnancy age, average annual household income, history of adverse pregnancy, pre-gestational diabetes, gestational diabetes, gestational hypertension, influenza before/during pregnancy, smoking before/during pregnancy, passive smoking before/during pregnancy, and alcohol intake before/during pregnancy.

**Table 3:** HWE of target loci of the *GATA4* gene in the control group.

Target Loci	MAF ^#^	Major Allele	Minor Allele	Genotype	Controls (*n*)	*χ* ^2^	*p*	*Q_FDR_*
rs4841588	0.325	T	G	TT/TG/GG	272/244/84	5.777	0.016	0.072
rs884662	0.282	T	C	TT/TC/CC	414/176/10	3.220	0.077	0.164
rs804287	0.250	C	T	CC/CT/TT	329/223/48	1.372	0.249	0.374
rs3203358	0.155	C	G	CC/CG/GG	558/42/0	0.789	0.631	0.811
rs867858	0.361	A	C	AA/AC/CC	242/204/154	55.834	<0.001	0.009
rs2645457	0.359	T	G	TT/TG/GG	576/24/0	0.250	1.000	1.000
rs10108052	0.379	A	G	AA/AG/GG	159/298/143	0.021	0.934	1.000
rs12458	0.400	G	A	GG/GA/AA	203/274/123	2.951	0.091	0.164
rs904018	0.368	A	G	AA/AG/GG	273/247/80	3.999	0.042	0.126

^#^ MAF: minor allele frequency, based on the global population of 1000 Genomes Project.

**Table 4:** Associations between polymorphisms of target loci at the *GATA4* gene and CHD.

Target Loci	CHD Cases (N = 585) (%)	Controls (N = 600) (%)	cOR (95% CI)	*p*	aOR (95% CI) ^#^	*p*
rs4841588						
TT	317 (54.1)	272 (45.3)	1.00	-	1.00	-
GT	239 (40.9)	244 (40.7)	0.84 (0.66–1.07)	0.157	0.98 (0.74–1.31)	0.909
GG	29 (5.0)	84 (14.0)	0.30 (0.19–0.47)	<0.001 *	0.41 (0.24–0.70)	<0.001 *
Dominant model	-	-	0.70 (0.56–0.88)	0.002 *	0.85 (0.65–1.12)	0.244
Additive model	-	-	0.66 (0.55–0.78)	<0.001 *	0.77 (0.62–0.95)	0.016 *
Recessive model	-	-	0.32 (0.21–0.50)	<0.001 *	0.41 (0.25–0.69)	<0.001 *
rs884662						
TT	408 (69.7)	414 (69.0)	1.00	-	1.00	-
CT	162 (27.7)	176 (29.3)	0.93 (0.73–1.20)	0.597	0.87 (0.65–1.18)	0.372
CC	15 (2.6)	10 (1.7)	1.52 (0.68–3.43)	0.310	1.89 (0.68–5.27)	0.225
Dominant model	-	-	0.97 (0.75–1.24)	0.781	0.91 (0.68–1.23)	0.551
Additive model	-	-	1.01 (0.81–1.26)	0.959	0.97 (0.75–1.27)	0.844
Recessive model	-	-	1.55 (0.69–3.49)	0.286	1.96 (0.71–5.46)	0.197
rs804287						
CC	344 (58.8)	329 (54.8)	1.00	-	1.00	-
TC	196 (33.5)	223 (37.2)	0.84 (0.66–1.07)	0.164	0.81 (0.61–1.09)	0.165
TT	45 (7.7)	48 (8.0)	0.90 (0.58–1.38)	0.622	0.99 (0.58–1.70)	0.972
Dominant model	-	-	0.85 (0.68–1.07)	0.168	0.84 (0.64–1.11)	0.217
Additive model	-	-	0.90 (0.75–1.08)	0.249	0.91 (0.73–1.13)	0.391
Recessive model	-	-	0.96 (0.63–1.46)	0.844	1.08 (0.64–1.83)	0.779
rs3203358						
CC	546 (93.3)	558 (93.0)	1.00	-	1.00	-
GC	39 (6.7)	42 (7.0)	0.95 (0.60–1.49)	0.820	1.07 (0.63–1.80)	0.814
Dominant model	-	-	0.95 (0.60–1.49)	0.820	1.07 (0.63–1.80)	0.814
Additive model	-	-	0.95 (0.60–1.49)	0.820	1.07 (0.63–1.80)	0.814
rs2645457						
TT	556 (95.0)	576 (96.0)	1.00	-	1.00	-
GT	29 (5.0)	24 (4.0)	1.25 (0.72–2.18)	0.425	1.27 (0.63–2.55)	0.500
Dominant model	-	-	1.25 (0.72–2.18)	0.425	1.27 (0.63–2.55)	0.500
Additive model	-	-	1.25 (0.72–2.18)	0.425	1.27 (0.63–2.55)	0.500
rs10108052						
AA	189 (32.3)	159 (26.5)	1.00	-	1.00	-
AG	249 (42.6)	298 (49.7)	0.70 (0.54–0.92)	0.010 *	0.86 (0.62–1.19)	0.356
GG	147 (25.1)	143 (23.8)	0.87 (0.63–1.18)	0.362	0.99 (0.68–1.44)	0.948
Dominant model	-	-	0.76 (0.59–0.97)	0.028 *	0.90 (0.66–1.22)	0.497
Additive model	-	-	0.92 (0.79–1.07)	0.289	0.99 (0.82–1.20)	0.907
Recessive model	-	-	1.07 (0.82–1.40)	0.604	1.09 (0.79–1.50)	0.608
rs12458						
GG	130 (22.3)	203 (33.8)	1.00	-	1.00	-
GA	281 (48.0)	274 (45.7)	1.60 (1.22–2.11)	<0.001 *	1.87 (1.34–2.60)	<0.001 *
AA	174 (29.7)	123 (20.5)	2.21 (1.61–3.04)	<0.001 *	2.20 (1.50–3.23)	<0.001 *
Dominant model	-	-	1.79 (1.38–2.32)	<0.001 *	1.98 (1.45–2.69)	<0.001 *
Additive model	-	-	1.49 (1.27–1.75)	<0.001 *	1.49 (1.23–1.81)	<0.001 *
Recessive model	-	-	1.64 (1.26–2.14)	<0.001 *	1.48 (1.08–2.04)	0.016 *
rs904018						
AA	333 (56.9)	273 (45.5)	1.00	-	1.00	-
AG	196 (33.5)	247 (41.2)	0.65 (0.51–0.83)	<0.001 *	0.80 (0.60–1.08)	0.144
GG	56 (9.6)	80 (13.3)	0.57 (0.39–0.84)	0.004 *	0.67 (0.42–1.06)	0.085
Dominant model	-	-	0.63 (0.50–0.80)	<0.001 *	0.77 (0.58–1.02)	0.064
Additive model	-	-	0.72 (0.61–0.85)	<0.001 *	0.81 (0.66–0.99)	0.046 *
Recessive model	-	-	0.69 (0.48–0.99)	0.043 *	0.73 (0.47–1.14)	0.171

cOR indicates crude odds ratios based on univariate analysis; aOR indicates adjusted odds ratios based on multivariable logistic regression. * *p* < 0.05; the difference between case and control is significant. ^#^ Adjusted for pregnancy age, average annual household income, history of adverse pregnancy, pre-gestational diabetes, gestational diabetes, gestational hypertension, influenza before/during pregnancy, smoking before/during pregnancy, passive smoking before/during pregnancy, and alcohol intake before/during pregnancy.

**Table 5:** Interactions between the polymorphisms of the *GATA4* gene and FA supplementation with CHD.

Target Loci	Took FA Supplementation before or during Pregnancy		Took FA Supplementation in the Preconception Period	
	aOR (95% CI) ^#^	*p*	aOR (95% CI) ^#^	*p*
rs4841588	0.60 (0.24–1.50)	0.277	0.87 (0.26–2.90)	0.824
rs884662	2.43 (0.98–6.01)	0.055	1.16 (0.35–3.85)	0.804
rs804287	0.45 (0.18–1.11)	0.084	0.40 (0.12–1.33)	0.133
rs3203358	1.12 (0.12–10.52)	0.921	1.96 (0.10–39.99)	0.663
rs2645457	NA	NA	NA	NA
rs10108052	4.94 (1.57–15.59)	0.006 *	3.20 (0.67–15.29)	0.146
rs12458	1.82 (0.69–4.85)	0.228	1.92 (1.54–6.92)	0.317
rs904018	0.70 (0.29–1.69)	0.422	0.58 (0.18–1.88)	0.363

NA: not available. * *p* < 0.05; the difference between the CHD cases and controls is significant. ^#^ Adjusted for pregnancy age, average annual household income, history of adverse pregnancy, pre-gestational diabetes, gestational diabetes, gestational hypertension, influenza before/during pregnancy, smoking before/during pregnancy, passive smoking before/during pregnancy, and alcohol intake before/during pregnancy.

## Data Availability

Data are unavailable due to privacy or ethical restrictions.
